# HER Specific TKIs Exert Their Antineoplastic Effects on Breast Cancer Cell Lines through the Involvement of STAT5 and JNK

**DOI:** 10.1371/journal.pone.0146311

**Published:** 2016-01-06

**Authors:** Daphne Gschwantler-Kaulich, Thomas W. Grunt, Daniela Muhr, Renate Wagner, Heinz Kölbl, Christian F. Singer

**Affiliations:** 1 Division of Gynecology, Department of OB/GYN and Comprehensive Cancer Center, Medical University of Vienna, Vienna, Austria; 2 Signaling Networks Program, Division of Oncology, Department of Medicine I and Comprehensive Cancer Center, Medical University of Vienna, Vienna, Austria; 3 Ludwig Boltzmann Cluster Oncology, Vienna, Austria; University of South Alabama, UNITED STATES

## Abstract

**Background:**

HER-targeted tyrosine kinase inhibitors (TKIs) have demonstrated pro-apoptotic and antiproliferative effects in vitro and in vivo. The exact pathways through which TKIs exert their antineoplastic effects are, however, still not completely understood.

**Methods:**

Using Milliplex assays, we have investigated the effects of the three panHER-TKIs lapatinib, canertinib and afatinib on signal transduction cascade activation in SKBR3, T47D and Jurkat neoplastic cell lines. The growth-inhibitory effect of blockade of HER and of JNK and STAT5 signaling was measured by proliferation- and apoptosis-assays using formazan dye labeling of viable cells, Western blotting for cleaved PARP-1 and immunolabeling for active caspase 3, respectively.

**Results:**

All three HER-TKIs clearly inhibited proliferation and increased apoptosis in HER2 overexpressing SKBR3 cells, while their effect was less pronounced on HER2 moderately expressing T47D cells where they exerted only a weak antiproliferative and essentially no pro-apoptotic effect. Remarkably, phosphorylation/activation of JNK and STAT5A/B were inhibited by HER-TKIs only in the sensitive, but not in the resistant cells. In contrast, phosphorylation/activation of ERK/MAPK, STAT3, CREB, p70 S6 kinase, IkBa, and p38 were equally affected by HER-TKIs in both cell lines. Moreover, we demonstrated that direct pharmacological blockade of JNK and STAT5 abrogates cell growth in both HER-TKI-sensitive as well as -resistant breast cancer cells, respectively.

**Conclusion:**

We have shown that HER-TKIs exert a HER2 expression-dependent anti-cancer effect in breast cancer cell lines. This involves blockade of JNK and STAT5A/B signaling, which have been found to be required for in vitro growth of these cell lines.

## Introduction

Members of the HER(ErbB) receptor tyrosine kinase family are known to be key modulators of breast cancer cell growth. They act mainly by promoting tumor cell proliferation and by inhibiting apoptosis, and because of their oncogenic role and their physical accessibility on the surface of the tumor cells, they represent important molecular targets for therapeutic intervention [1,2]. Indeed, several HER-targeting antibodies and small molecule-type inhibitors (TKIs) have been developed, and the HER2-specific antibody trastuzumab has profoundly improved treatment outcome in HER2 overexpressing human breast cancer [3]. Nevertheless, a substantial fraction of patients with HER2 overexpressing tumors progress despite initial response. Interestingly, up to 30% of trastuzumab-resistant patients are at least temporarily responsive to the reversible HER1/2 inhibitor lapatinib [4]. This suggests that direct blockade of both the EGFR (HER1, ErbB1) and the HER2/neu (ErbB2) tyrosine kinase domains by the kinase inhibitor is more effective than interference of the antibody with the extracellular domain of HER2 only. Moreover, retention of sensitivity of trastuzumab resistant cells against the HER1/2 dual inhibitor also suggests that additional pro-oncogenic signaling pathways are being activated [5]. Although the exact mechanisms of resistance against HER2-specific targeting remain unknown, it has been suggested that heterodimerization and cross-activation of HER2 by other members of the HER family (including EGFR/HER1, ErbB3/HER3 and ErbB4/HER4) does occur. This cross-talk could potentially be overcome by using pan-HER inhibitors. Intriguingly, several pan-HER targeting drugs have shown promising in vitro activity even in HER2 negative tumors suggesting a HER2-independent effect on HER family members and on cancer cell growth [6–10]. A better understanding of the molecular mechanisms by which HER-specific TKIs exert their inhibitory effects on tumor cell growth and survival is thus essential for the improvement of the therapy of HER2 overexpressing breast cancer.

In principle, HER-dependent signaling acts on cell proliferation and protein synthesis via 2 pathways: Through the phosphatidylinositol 3-kinase (PI3K) and its mediators AKT, mTOR and p70 S6 kinase, and through the mitogen-activated protein kinase (MAPK) cascade-related proteins c-RAF, MEK, and ERK 1/2 [11–15]. The HER family also controls tumor cell proliferation and apoptosis through separate, less known pathways. These pathways involve STAT5A/B, p38 and JNK, which regulate caspase activation and PARP-1 cleavage via BCL2 [16,17]. Surprisingly, the effect of pan-HER-targeted TKIs on these pathways has never been evaluated, even though modulation of proliferation and control of cell death are essential for the fate of a malignant breast tumor. We have therefore investigated the effects of the reversible HER inhibitor lapatinib and of the two novel potent irreversible HER-targeting drugs afatinib (BIBW 2992) and canertinib (CI-1033) on downstream modulators of proliferation (STAT5, ERK 1/2, STAT3, CREB, p70 S6 kinase), and apoptosis (IkBa, p38 and JNK).

Lapatinib is a small, reversible, dual inhibitor of EGFR (HER1) and HER2. It has demonstrated potent antitumor effects in HER2 overexpressing models in vitro, including cell lines with acquired trastuzumab resistance [4,5]. Several phase III studies have now also confirmed the efficacy of lapatinib in the metastatic setting when given in combination with either chemotherapy or aromatase inhibitors. Canertinib (CI-1033) is an irreversible inactivator of HER1, HER2 and HER4, which has also demonstrated efficacy in early-phase trials of advanced solid tumors and HER2 positive metastatic breast cancer [7,18–21]. Afatinib (BIBW 2992) is another irreversible, oral pan-HER inhibitor, which has demonstrated activity in early-phase trials of advanced solid tumors and trastuzumab-refractory HER2-positive breast cancer [22]. In order to account for potential differences in HER-specific effects, all three HER-TKIs were tested on cell lines, which reveal different degrees of HER2 expression ranging from no expression (Jurkat) to moderate (T47D) and high levels of HER2 (SKBR3).

## Materials and Methods

### Reagents

Lapatinib was purchased from ChemieTek (Indianapolis, IN), afatinib (BIBW 2992) was from Selleckchem (Houston, TX), and canertinib (CI-1033, PD-183805) was kindly provided by Pfizer (Groton, CT), respectively. All kinase blockers were dissolved in DMSO and diluted 1:1,000 or 1:2,000 in medium before use. Recombinant epidermal growth factor (EGF) and heregulin-b1 (HRG-b1) were purchased from Sigma (St. Louis, MO). Fetal calf serum (FCS), DMEM, a-MEM, and RPMI 1640 were from GIBCO (Karlsruhe, Germany). The JNK inhibitor SP600125 and the STAT5 inhibitor II (IQDMA) were obtained from Selleckchem and from Merck (Darmstadt, Germany), respectively.

### Cell lines

The HER2-overexpressing SKBR3 and the HER2-moderately expressing T47D human breast cancer cell lines were purchased from the American Tissue Type Culture Collection (Gaithersburg, MD), respectively. The HER-negative human leukemia-derived cell line Jurkat was kindly provided by U. Jäger (Medical University of Vienna, Austria). Cells were maintained in DMEM, a-MEM, or RPMI 1640 medium with 10% FCS, antibiotics, and 2mM glutamine (GIBCO). Cells were cultured for up to 20 passages. All three cell lines were tested for the absence of mycoplasma (Venor GeM, Minerva Biolabs, Berlin, Germany). Cell morphology and expression of marker antigens (HER, estrogen receptor, retinoic acid receptors, and fatty acid synthase) were checked to exclude cross-contamination of cell lines.

### Cell growth assays

Cells were plated at 1.5x10^3^/well in 96 well culture plates. After adhesion, the HER-, JNK-, or STAT5-inhibitors were added at various concentrations in 5% FCS in the absence of EGF and HRG-b1. After 72h, cell numbers were determined using a formazan dye assay (Biomedica, Vienna, Austria). The cell numbers were plotted against the concentration of each drug. Sigmoidal curves were calculated with the Boltzmann equation using GraphPad Prism 4.03 software and the drug concentrations leading to 50% reduction of the cell numbers (IC_50_) were obtained from these curves as described previously [23].

### Western blotting

Cells plated in media containing 5% FCS at 5 x 10^5^ in 60 mm dishes were allowed to adhere and were exposed for 48 or 72 hours to various concentrations of canertinib. Cells were then lysed, and protein fractions (30 μg/lane) were subjected to SDS-PAGE, blotted onto PVDF membranes, and immunostained as described [24] using antibodies against full length and cleaved PARP-1 (Cell Signaling Technology, Boston, MA) or against actin (I-19, Santa Cruz Biotechnology, Santa Cruz, CA) at 1:1,000. Membranes were then incubated with peroxidase-tagged secondary antibodies. Antibody binding was visualized by enhanced chemiluminescence and quantitated by scanning densitometry of the obtained autoradiographs using ImageJ software (National Institutes of Health, Bethesda, MD). Actin was used as loading control. However, since PARP-1 and Actin were on different membranes, quantitative estimation of PARP-1 cleavage was done by calculating the ratio between the optical density (OD) of cleaved PARP-1 band at 89 kDa and the full-length PARP-1 band at 116kDa (OD_cleavedPARP-1_/ OD_full-length PARP-1_).

### Milliplex assays

We used the Multiplexed immunoassay „Multi-Pathway-8Plex”from Millipore and performed the assays according to the array protocol.

Briefly, 24 h after seeding, the cells were depleted from serum for another 24 h, and then exposed for 2 or 6 h to 1 μM HER-TKIs. Before lysis in Milliplex MAP Lysis Buffer, the cells were challenged for 3 min with 100 ng/ml EGF and 10 nM HRG-b1 and prepared for subsequent analysis.

### Statistical analyses

The Student´s t test for unpaired samples was used to examine the significance in differences of growth in drug-treated cells. P-values ≤ 0.05 were considered significant.

## Results

**Fig 1:**
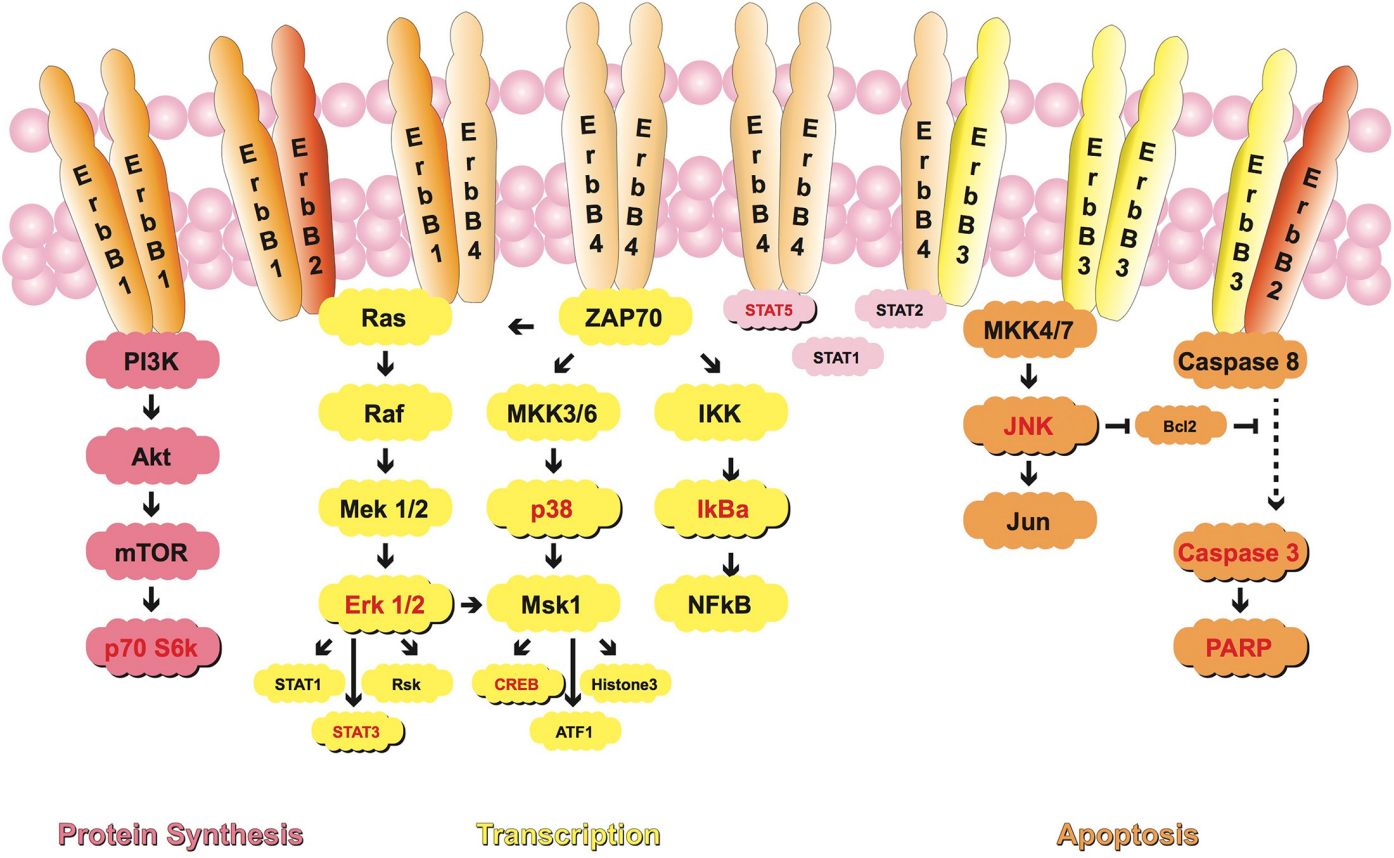
Schematic representation of HER-dependent signal transduction pathways associated with cell proliferation and apoptosis.

Fig 1 shows the pathways involved in cell proliferation and apoptosis and the investigated parameters.

### Differential growth-inhibitory effects of lapatinib, afatinib and canertinib are dependent on the degree of HER2 expression

**Fig 2:**
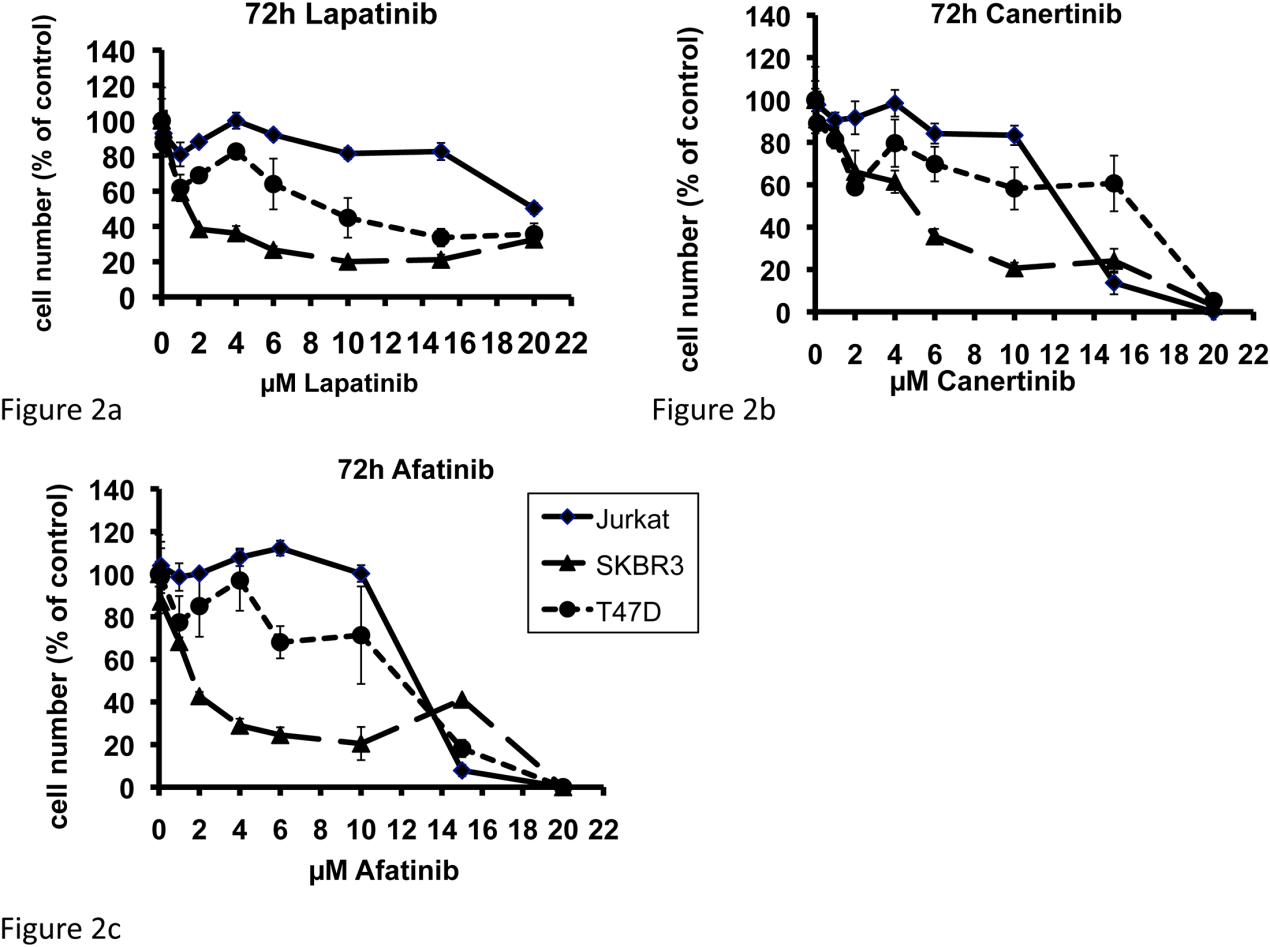
Dose-dependent growth inhibition in response to 72h exposure to lapatinib (a), canertinib (b), or afatinib (c) on malignant human cell lines with no HER2 expression (Jurkat), with moderate HER2 expression (T47D) and with HER2 overexpression (SKBR3). Means ± SD, n = 3.

The growth-inhibitory effects of lapatinib, afatinib, and canertinib on malignant human cell lines with differential HER2 protein expression are shown in Fig 2. Cell lines with no HER2 expression (Jurkat), with moderate HER2 expression (T47D) and with HER2 overexpression (SKBR3) were cultured for 72h with various concentrations of the respective HER-TKI, and the IC_50_ values were determined. Lapatinib inhibited the HER2 overexpressing cell line SKBR3 at an IC_50_-concentration of 1.444μM and was significantly more effective in this cell line than in the moderately HER2 expressing cell line T47D (IC_50_ = 9.082μM) and the HER2 negative cell line Jurkat (IC_50_: 20.091μM) (Fig 2a). Comparable effects were observed with afatinib, which was also more effective in inhibiting tumor cell growth in SKBR3 cells (IC_50_: 1.708μM) than in T47D (IC_50_ = 11.422μM) and in Jurkat cells (IC_50_ = 12.718μM) (Fig 2b). Canertinib blocked SKBR3 cells at an IC_50_ of 4.873μM while the inhibitory effect was again much weaker in T47D (IC_50_ = 15.804μM) and in Jurkat cells (IC_50_ = 12.397mM) (Fig 2c). Overall, the antiproliferative effect of each of the three HER-TKIs was reached in a significantly lower dosage range in HER2 overexpressing cells (SKBR3) than in cells with moderate (T47D; p = 0.014, unpaired t-test) or no HER2 expression (Jurkat; p = 0.011, unpaired t-test). The three TKIs did not differ significantly in their growth-inhibitory effect in any of the cell lines analyzed (lapatinib versus afatinib, p = 0.82; lapatinib versus canertinib, p = 0.85; afatinib versus canertinib, p = 0.66).

### Effect of lapatinib, afatinib and canertinib on the inhibition of HER downstream signal transduction pathways

**Fig 3:**
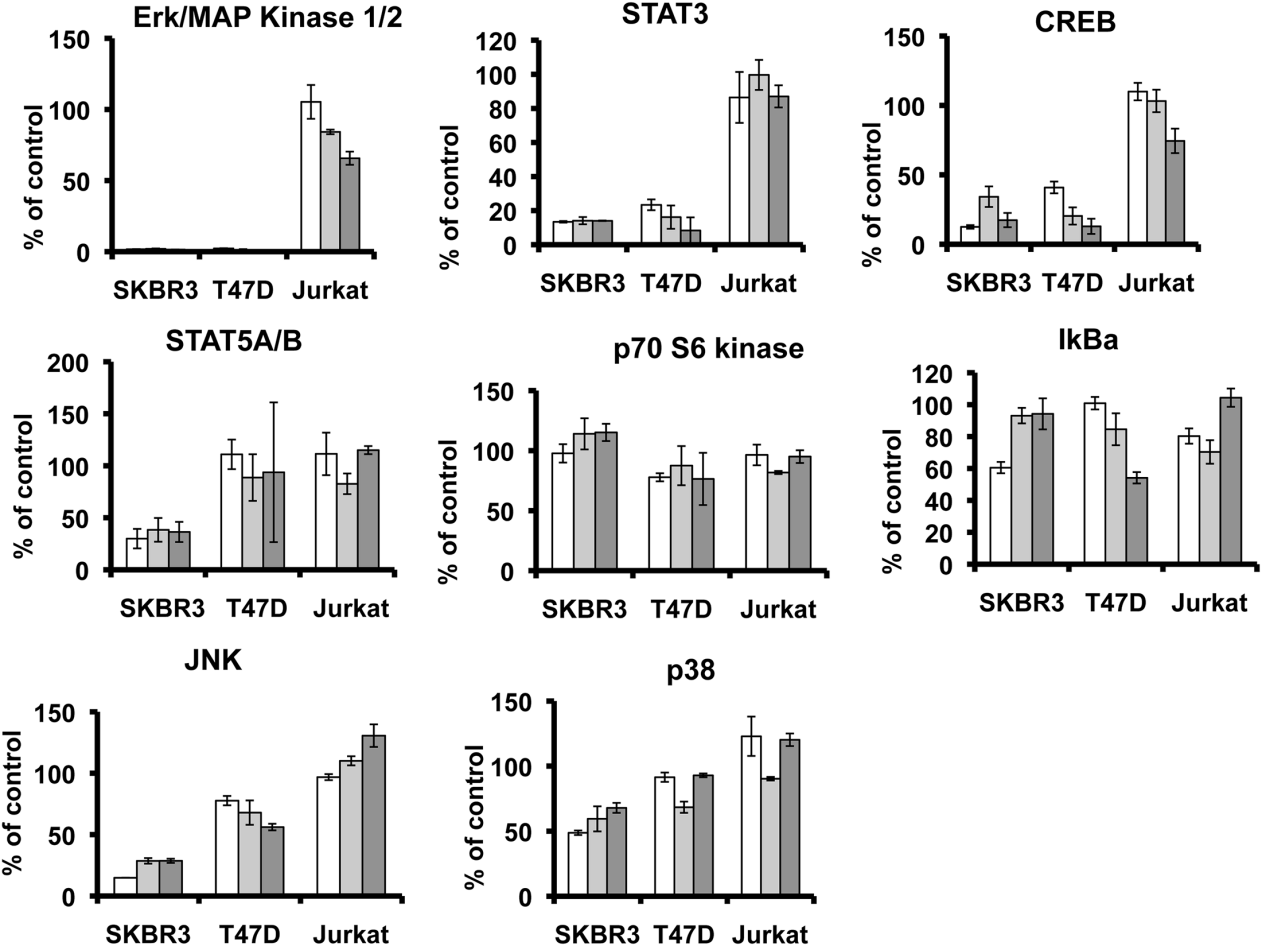
Effect of canertinib (white bar), lapatinib (light grey bar) and afatinib (dark grey bar) on activation (phosphorylation) of ERK/MAPK1/2, STAT3, CREB, STAT5A/B, p70 S6 kinase, IkBa, JNK, and p38 in SKBR3, T47D and Jurkat cells. Serum-depleted cells were exposed for 6 h to vehicle (0.1% DMSO) or to 1 μM of the TKIs followed by a 3-min challenge with 100 ng/ml EGF and 1 nM HRGb1 and subjected to Milliplex assays. Results are shown relative to vehicle control (100%). Means ± SD.

We then examined the effect of the three HER-TKIs on the inhibition of proliferation- and apoptosis-associated downstream effector pathways in SKBR3, T47D and Jurkat cells by using phosphotyrosine-specific Milliplex assays (Fig 3). Lapatinib, afatinib and canertinib profoundly inhibited the phosphorylation of the proliferation-associated signaling effectors ERK/MAPK (p<0.0001, and p<0.0001, respectively, Student´s t test), STAT3 (p<0.0001, and p<0.0001, respectively) and CREB (p<0.0001, and p<0.0001, respectively) in both HER2 overexpressing SKBR3 and moderately expressing T47D cells, but not in HER2 negative Jurkat cells (Fig 3). Phosphorylation of STAT5A/B was only inhibited in TKI-sensitive SKBR3 cells (p<0.0001), but not in TKI-resistant T47D (p = 0.07) or Jurkat cells (p = 0.41) (Fig 3). Moreover, a distinct and HER2 expression-related pattern of responsiveness against the three HER-TKIs was observed for the phosphorylation/activation of HER2-dependent and apoptosis-related stress proteins SAPK/JNK and p38. Particularly, phosphorylation of JNK was profoundly inhibited by all three HER-TKIs in the HER2 overexpressing sensitive SKBR3 (p<0.0001), whereas the effect was either less pronounced (p = 0.0002) or completely absent (p = 0.06) in the moderately HER2 positive resistant T47D cells and the HER2 negative Jurkat cells, respectively. A similar, albeit weaker pattern of distinctive responsiveness was seen for p38 (p<0.0001, p = 0.008, and p = 0.14). On the other hand, there was no consistent inhibitory effect of the HER-TKIs on the proliferation-associated downstream effectors p70 S6 kinase and IkBa in the three cell lines examined (Fig 3).

### The inhibitory effect of HER-TKIs on cell proliferation involves diminished phosphorylation/activation of JNK and STAT5A/B

**Fig 4:**
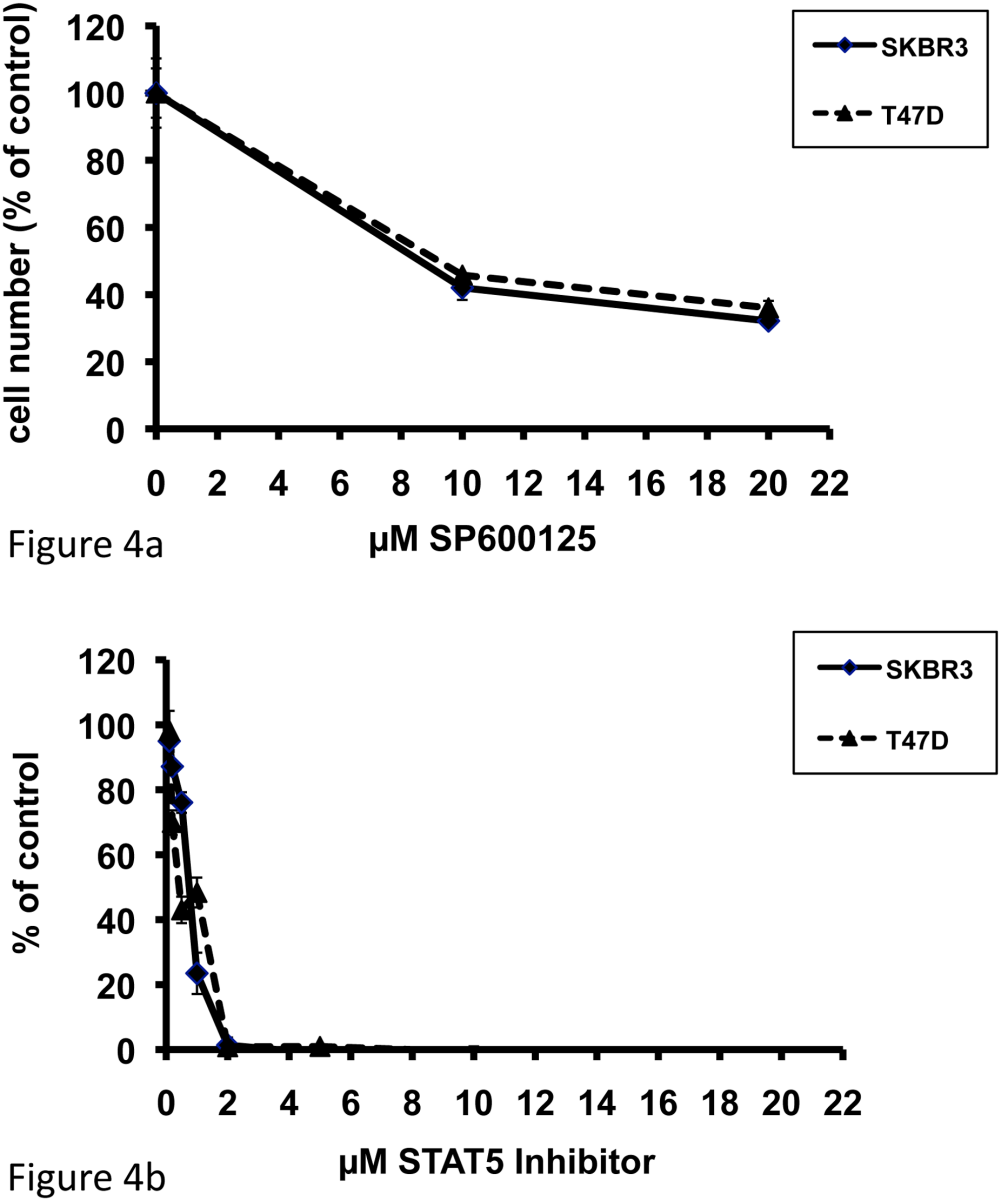
Dose-dependent growth inhibition in response to 72h exposure to the JNK inhibitor SP600125 (a) or the STAT5 Inhibitor II IQDMA (b) on SKBR3 and T47D human breast cancer cells. Means ± SD.

In order to examine the relationship between HER-TKI-dependent blockade of JNK and STAT5A/B and the growth-inhibitory action of the HER-inhibitors, SKBR3 and T47D cells were treated with SP600125, a highly specific anthrapyrazolone inhibitor of JNK, or with the synthetic STAT5 inhibitor II (IQDMA). SP600125, when applied at 10 and 20 μM for 72h, exerted a dose-dependent and significant reduction in cell numbers, which was identical in both cell lines (Fig 4a). In analogy, IQDMA, when added at concentrations ranging from 0.1 to 20 μM, exerted a dose-dependent inhibition of tumor cell growth and resulted in a complete cell loss at a concentration of 2 μM in both SKBR3 and T47D cells (Fig 4b). These results indicate that breast cancer cells require activation of JNK and STAT5A/B for growth and that this activity is lost in breast cancer cells that are sensitive to HER-TKIs. The data therefore suggest that phospho-JNK and/or phospho-STAT5A/B levels may be used as predictors for HER-TKI sensitivity of breast cancer cells.

### Pan-HER-TKIs promote apoptosis in HER2 overexpressing tumor cells through HER-mediated pathways

**Fig 5:**
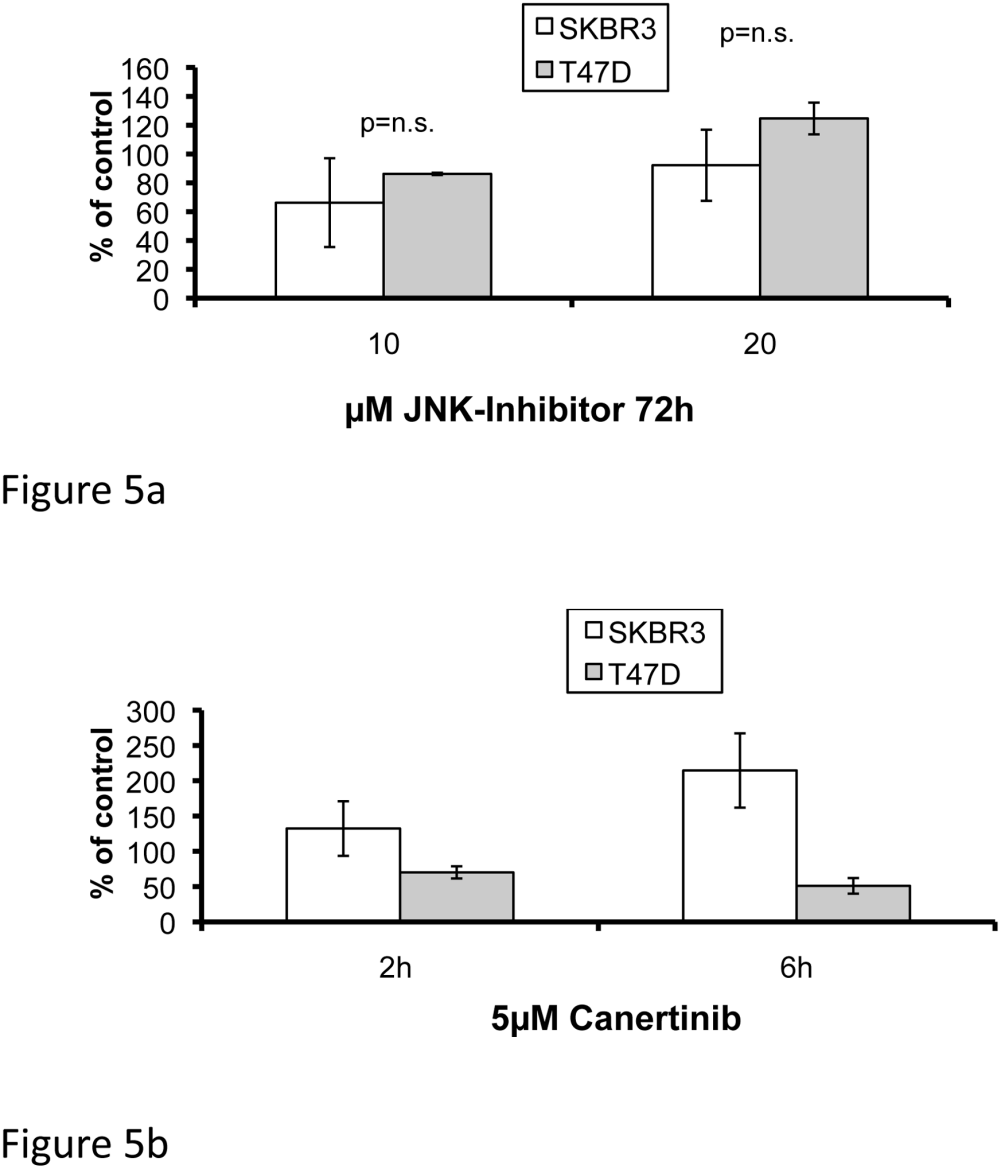
Dose-dependent effect of SP600125 when given for 72h (a) and time-dependent effect of 5μM canertinib after 2 and 6 hours (b) on apoptosis as examined by Milliplex immunoassay for detection of active (cleaved) caspase 3 in SKBR3 and T47D cells. Results are shown relative to vehicle (0.1% DMSO) control. Means ± SD.

**Fig 6:**
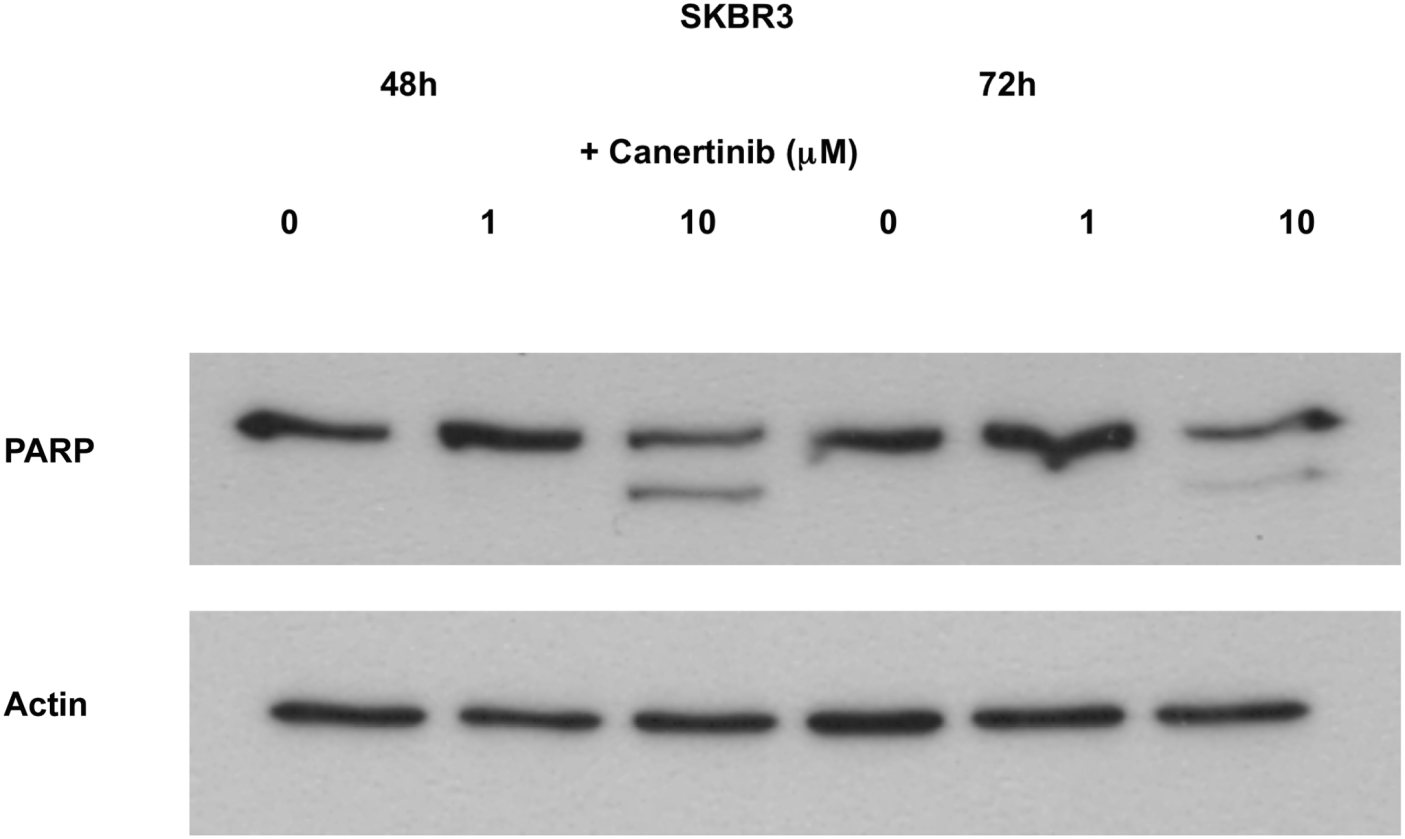
Effect of the HER-TKI canertinib on the expression of apoptosis-related PARP-1 cleavage products as demonstrated by Western blot analysis of SKBR3 cells grown for 48h (lanes 1–3) and 72h (lanes 4–6) in the absence (lanes 1 and 4), or in the presence of 1μM (lanes 2 and 5) and 10 μM (lanes 3 and 6) canertinib. Actin was probed on a separate membrane to roughly estimate protein loading.

**Fig 7:**
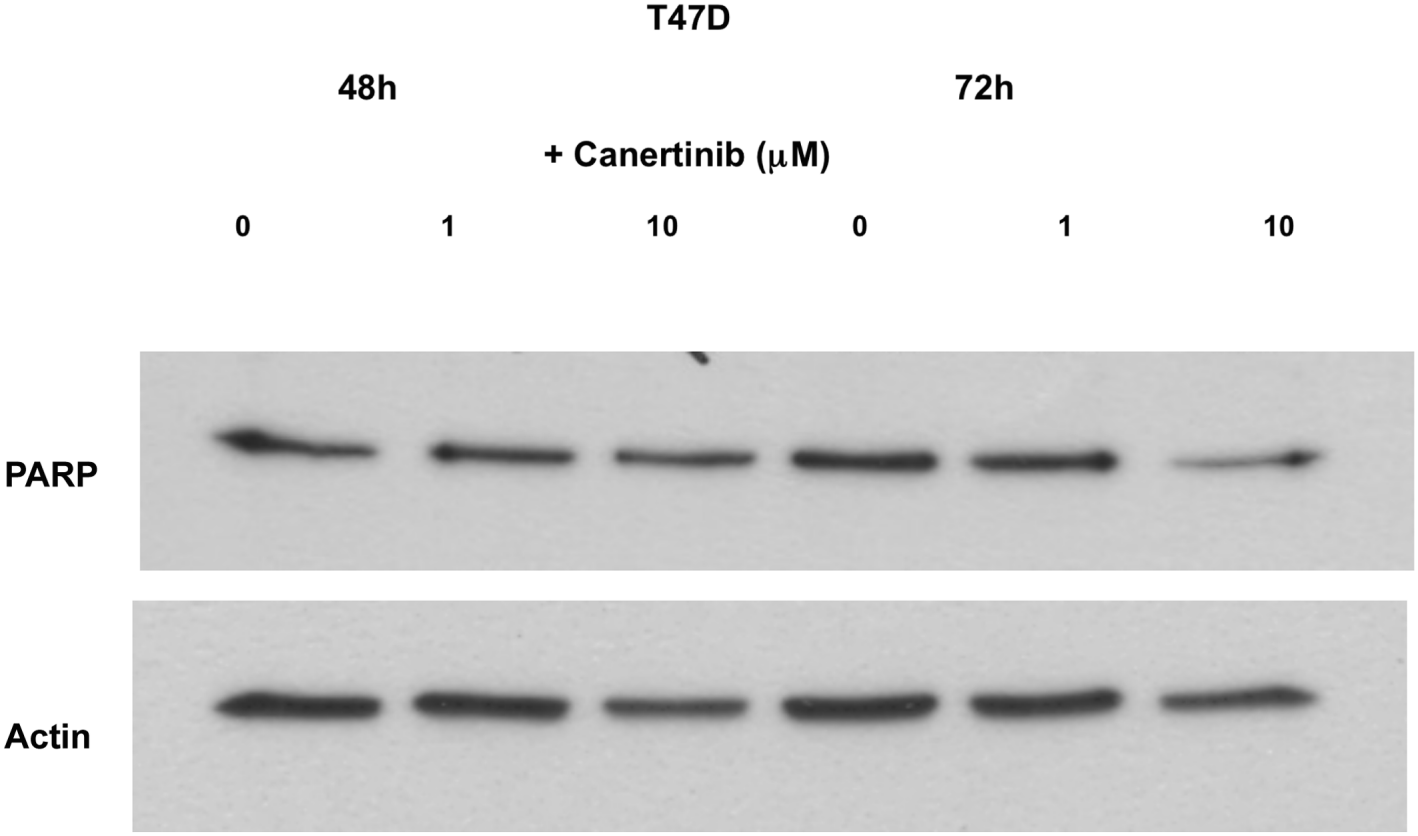
Effect of the HER-TKI canertinib on the expression of apoptosis-related PARP-1 cleavage products as demonstrated by Western blot analysis of T47D cells grown for 48h (lanes 1–3) and 72h (lanes 4–6) in the absence (lanes 1 and 4), or in the presence of 1μM (lanes 2 and 5) and 10 μM (lanes 3 and 6) canertinib. Actin was probed on a separate membrane to roughly estimate protein loading.

Detection of active caspase 3 applying Milliplex technology was used to determine the pro-apoptotic activity of direct inhibition of JNK or HER, respectively. Blockade of JNK with SP600125 at concentrations of 10μM and 20μM for 72 hours did not induce active caspase 3 in either of the two cell lines tested (Fig 5a) suggesting that the anticancer effect of SP600125 was mediated rather by growth arrest than by apoptosis. In contrast, depending on cellular HER2 expression, pan-HER-TKIs induced a differential apoptotic response. Accordingly, when SKBR3 cells were exposed to canertinib, a significant and time-dependent increase in active caspase 3 was observed in response to 5μM canertinib (6 vs. 2 hours, p = 0.03), whereas no response was seen in T47D cells (6 vs. 2 hours, p = n.s.) (Fig 5b). These data were corroborated by experiments using Western blotting for detection of PARP-1 cleavage products, which represent biomarkers for apoptotic cell death: apoptosis-associated PARP-1 cleavage products were observed in SKBR3 but not in T47 cells after 48 and 72h of 10μM canertinib treatment (Figs 6 and 7). Accordingly, after densitometric scanning of the bands using ImageJ software, the calculated ratios of the optical density values (OD) between cleaved PARP-1 and full-length PARP-1 yielded zero for all conditions in both cell lines except in SKBR3 treated with 10μM canertinib for 48h (OD_cleavedPARP-1_/OD_full-length PARP-1_ = 0.484) or 72h (OD_cleavedPARP-1_/OD_full-length PARP-1_ = 0.139), respectively. Thus, our data indicate that the pro-apoptotic effect of the pan-HER-TKIs is indeed HER-mediated and not the result of a direct inhibition of JNK.

## Discussion

Members of the HER family of transmembrane receptor tyrosine kinases have been implicated in several aspects of breast cancer development and tumor progression. Among them, HER2 has gained particular interest, because HER2 overexpression is present in approximately 20% of invasive breast cancer cases and renders these tumors responsive to treatment with the monoclonal antibody trastuzumab (Herceptin^®^). However, the fact that the majority of initially responding breast cancers progress during antibody treatment, and the notion that other members of the HER family can cross-activate downstream signal transduction, has led to the development of TKIs with a broader target specificity.

In contrast to earlier developments such as gefitinib (Iressa, ZD1839) and erlotinib (Tarceva, OSI-774), which preferentially inhibit EGFR/HER1 signaling, lapatinib (Tykerb, GW572016) was the first clinically evaluated TKI, which inhibits the growth of both EGFR/HER1 and HER2 overexpressing cells. It is a reversible inhibitor with an IC_50_ of 10.8 nM for EGFR/HER1 and 9.2 nM for HER2, respectively. It also weakly inhibits HER4 with an IC_50_ of 367 nM, but has a more than 300-fold selectivity for HER family members over other kinases such as c-Src, c-Raf, MEK, ERK, or p38 [4]. Although being initially effective even in trastuzumab-resistant tumors, subsequent progression towards resistance to these reversible first generation pan-HER inhibitors has led to the development of second generation irreversible pan-HER-TKIs such as afatinib and canertinib.

Afatinib (Tomtovok, BIBW 2992) is an oral, HER family blocker, which irreversibly blocks all kinase-competent HER members through covalent receptor binding with IC_50_ values of 50 nM for HER1, of 14 nM for HER2 and of 1 nM for HER4, respectively [25]. In a single arm open-label phase II study Lin et al. have explored afatinib activity in HER2 overexpressing patients who had progressed after trastuzumab treatment and found promising clinical activity [26]. Interestingly, the drug has also shown promising activity in triple-negative xenograft models of breast cancer. However, a recently conducted phase II trial in HER2 negative breast cancer indicated only limited activity, which supports our clinical finding of a significant association between the antiproliferative effect of HER-TKIs and HER2 expression [23]. Canertinib (CI-1033, PD-183805) has somewhat different binding affinities with IC_50_ of 5, 14 and 10 nM for EGFR/HER1, HER2 and HER4, respectively [27]. It has been evaluated in a phase II trial in heavily pretreated patients with HER2 +, ++, and +++ metastatic breast cancer. Not surprisingly, single-agent canertinib did not show clinically meaningful activity in this setting, presumably since patients with non-HER2 overexpressing tumors were also included in the trial [21]. While the authors do not present separate response rates for HER2+++ vs. HER2+ and ++ tumors, it is well possible that just like in our SKBR3 model, the antitumoral effect in HER2+++ tumors can be achieved within a pharmacological range while in HER2+ and ++ the weak antiproliferative effect of TKIs would require far higher drug concentrations, which would consequently result in unacceptable toxicity [28].

The above cited clinical observations and our own results shown here are, however, somewhat contrasted by recent findings by Trinks et al., who reported that canertinib displays anti-proliferative and pro-apoptotic effects in HER2 devoid Jurkat cells, which we have used as negative control cells [23]. They showed that canertinib was able to act as a multi-kinase inhibitor, which also affects Akt and ERK1/2. In their hands, treatment with canertinib at increasing concentrations induced PARP-1 cleavage and activated caspase 3, 8, 9 and 10 protein in a time-dependent manner, thereby indicating a pro-apoptotic effect. They suggest that even in HER2 negative cells canertinib is able to both downregulate proliferative signaling and activate apoptosis, although no comparison to HER2-overexpressing breast cancer cells has been provided.

Despite considerable differences in pharmacokinetics, binding reversibility and respective IC_50_ values, we did not observe differential effects between the three HER-TKIs in respect to the activity of selected downstream signal transduction proteins, cell proliferation, or apoptosis.

We observed that ERK/MAPK, STAT3 and CREB were inhibited in both sensitive and resistant breast cancer cell lines, whereas JNK and STAT5A/B were only inhibited in the sensitive cell line SKBR3, which suggests that in addition to hyperactivity of HER and of downstream PI3K signaling [24] these two parameters also contribute to the development of resistance of breast cancer cells against HER-TKIs. This hypothesis is supported by studies in pancreatic and prostate cancer, which also suggest a role for JNK activation in the development of resistance to HER targeting [16,17,29–31]. Moreover, EGF- and HRG-b1-induced phosphorylation of STAT5 has similarly been shown to modulate HER signaling and may thus also affect HER-TKI efficacy [32–36].

In summary, our data show that lapatinib, afatinib and canertinib exert HER2-dependent effects on proliferation and apoptosis in vitro. While several of the investigated pathway proteins including ERK1/2, STAT3, CREB, p70 S6 kinase, and IkBa did appear to be regulated independent from the level of HER2 expression and were not affected by TKI-mediated HER receptor blockade, JNK and STAT5A/B clearly showed differential response against the HER-TKIs, which directly correlated with the sensitivity of the breast cancer cells against these TKIs. Thus, silencing of JNK and STAT5A/B appears to be involved in the anticancer effects of the HER-TKIs and inactivation of these signal effector molecules could therefore potentially serve as biological indicator for sensitivity of breast cancer cells against HER-TKIs.
